# Roles of role players in the implementation of school-based human immunodeficiency virus and acquired immunodeficiency syndrome prevention programmes in local high school settings

**DOI:** 10.4102/hsag.v25i0.1301

**Published:** 2020-06-04

**Authors:** Constance B. Sekgobela, Doriccah Peu, Maretha de Waal

**Affiliations:** 1 Department of Health Studies, University of Pretoria, Pretoria, South Africa; 2 Department of Nursing Science, University of Pretoria, Pretoria, South Africa

**Keywords:** role players, implementation, school-based HIV and AIDS prevention programmes, reproductive health services, social ecological model

## Abstract

**Background:**

Worldwide, a large proportion of all new HIV infections occur in people under the age of 25. HIV and AIDS remain the leading cause of deaths among adolescents in sub-Saharan Africa and second leading cause of death globally. Preventing new HIV infections and AIDS-related deaths are at the heart of South Africa’s National Strategic Plan on HIV and AIDS, STIs and TB 2017-2022.

**Aims:**

Explore and describe the roles of the local role players in the implementation of the school-based HIV and AIDS prevention programmes in local high school settings.

**Settings:**

The study was conducted in the Bushbuckridge local municipal area in Mpumalanga Province.

**Methods:**

A qualitative, explorative, descriptive design and contextual study was used. Individual interviews and focus group interviews were conducted with the purposively selected participants from the clinic, health centre, high schools and community members. Data was analysed using Tesch’s method of data analysis.

**Results:**

Although all the local role players were found to have important roles to play in the implementation of the school-based HIV and AIDS prevention programme, gaps exist in the rendering of youth friendly services (YFS); accessible clinic times; HIV and AIDS education; life skills education; Life Orientation (LO) and health education; information sessions; counselling; school health programmes; campaigns as well as collaborative working strategy.

**Conclusion:**

Successful development and collaborative implementation of the school-based HIV and AIDS prevention programme can result in significant changes in knowledge and attitudes that affect sexual behaviour of young people, leading to significant decrease in HIV infection among young people.

## Introduction

According to the Joint United Nations Programme on HIV/AIDS (UNAIDS 2016) and World Health Organization (WHO 2016, 2017), adolescents and youths have the highest incidences of human immunodeficiency virus (HIV) infection and acquired immunodeficiency syndrome (AIDS). In 2016, it was estimated that 36.7 million people were living with HIV and AIDS worldwide, of whom 1.8 million were children. The prevalence of HIV amongst adults was 0.8% (Kaiser Family Foundation 2017:1; UNAIDS 2017). Around 30% of such people did not know that they were living with HIV. Also, in 2016, there were approximately 1.8 million new HIV infections, which was a decline from the 2.1 million new infections reported in 2015 (UNAIDS 2017). In spite of this decline, much more needs to be performed to improve the knowledge of HIV as well as HIV testing amongst adolescents and the youth. Indeed, various HIV and AIDS prevention programmes have proven to effectively reduce the infection worldwide. However, such prevention programmes have not reduced the infection rate in South Africa, especially amongst the youth. The WHO (2016) associated an increase in HIV infection among women aged between 15 and 24 years with various social factors as well as insufficient access to education and sexual and reproductive health services (SRHs). According to UNAIDS and United Nations International Children’s Emergency Fund (UNAIDS UNICEF 2016): ‘At the current pace, a projected 35 000 adolescents would die from AIDS-related illnesses in 25 countries in 2020’, and South Africa is among these 25 countries. Therefore, there is a need for HIV and AIDS health promotion interventions that specifically support reduction of new infections amongst the youth. Identifying the roles that each role player has to play for implementing the school-based HIV and AIDS prevention programmes may be vital for their success, ultimately leading to reduction of fresh infections amongst the youth.

In South African public schools, HIV and AIDS prevention programmes have been offered as part of the life orientation (LO) school curriculum (Department of Education [DoE] 2002b; Fatoba 2013; International Bureau of Education [IBE] 2012; IRIN/PlusNews Report 2008; Mukoma et al. 2009). Life orientation, life skills and sex education have been ongoing in South African schools for quite a while; however, it has been observed that there are challenges in meeting the objectives of HIV and AIDS education programmes. Some of the role players are not involved in the provision of HIV and AIDS services, and provision of health education thereof. According to South Africa’s National Strategic Plan for HIV, tuberculosis (TB) and sexually transmitted infections (STIs) 2017–2022, there are calls for implementing social and behavioural change programmes to address key drivers of the epidemic and build social cohesion. This could be achieved through strong collaboration between the Department of Social Development (DSD), Department of Basic Education (DBE) and Department of Health (DoH) (DSD/DBE/DoH) to ensure continuum of care for teenage mothers and their families, and strengthening of the Integrated School Health Programme (South African National AIDS Council [SANAC] 2017).

Nursing services as well as local key role players are fundamentals for the prevention and alleviation of the impact of HIV and AIDS. The Minister of Health, Dr Aaron Motsoaledi, was quoted highlighting the indispensable role of nurses in the fight against HIV and AIDS Mkhize (2013):

The nursing services are the heartbeat of primary healthcare, it might be easy to forget that the nursing fraternity helped achieve something we couldn’t have in 3 years – (increasing) life expectancy from 56 to 60 years. People may wonder what it has to do with nursing, but in terms of expanding HIV programmes, we couldn’t have done it without nurses.

The minister might be true with regard to the success of school HIV and AIDS prevention programmes. The Integrated School Health Policy (ISHP) is the result of a collaboration between the DoH and DBE (DoH and DBE 2012). The ISHP aims at building on and strengthening the existing school health services in collaboration with all role players (DoH & DBE 2012).

Proper implementation of key strategies in the ISHP and the full participation of various role players involved in the school-based HIV and AIDS prevention programmes are critical for their success. This may further contribute to the achievement of Sustainable Development Goals (SDGs) targets, particularly SDGs 1, 2 and 3 (Kutesa 2015). The social-ecological model was used as a framework to explore and further discuss findings in relation to the multiple effects and interrelatedness of social elements in school-based HIV and AIDS prevention programmes. It is in relation to this background that this study focusses on exploring the functions of role players in implementing school-based HIV and AIDS prevention programmes.

## Research methods and design

### Study design

A qualitative, explorative and descriptive design contextual study that was conducted was used to allow the researcher to interact with participants to gain a deeper understanding of the phenomenon being studied and to be able to find ways to address an issue or find solutions to problems (Grove, Burns & Gray 2013). The roles of the local role players in implementing the school-based HIV and AIDS prevention programmes were explored and described.

### Settings

The study was conducted in the Bushbuckridge local municipality of the Mpumalanga Province. Bushbuckridge municipality is a category B municipality that forms part of the five local municipalities of the Ehlanzeni District municipality family in the province. The municipality has three hospitals, 34 clinics, five mobile clinics and two community health centres (CHC) servicing the entire municipal area. Also, there are 213 primary schools, 199 high schools with combined schools and four Further Education and Training (FET) institutions. The choice of settings was informed by the HIV prevalence levels; Mpumalanga is the province with the second highest HIV levels – which is over 20%. Mpumalanga has a prevalence rate of 34.6% following KwaZulu-Natal with a prevalence rate of 37.4%. Bushbuckridge has a higher HIV rate than all other municipalities in the Mpumalanga Province (Fokazi 2012; Shisana et al. 2014; Van der Linde 2013), with the highest youth population as well as differences in infrastructure and wealth in the two townships identified for the study.

### Study population and sampling

The study population included local role players who had a designated responsibility in school health teams in the Bushbuckridge area of Mpumalanga. The role players comprised members of the school governing body (educators and parents), non-governmental organisations (NGOs), local clinics’ primary healthcare (PHC) nurses and the district health manager. Purposive sampling was used to select population participants that could provide rich and in-depth information (Grove et al. 2013; LoBiondo-Wood & Haber 2010). The CHC, clinic operational managers and senior members of high schools were requested to purposively identify participants with the knowledge and experience to provide rich and in-depth information.

### Data collection

Data were collected through semi-structured focus group interviews (FGIs) and semi-structured individual interviews by the researcher. Focus group interviews were carried out to elicit textual and structural descriptions of the experiences of local role players as well as to provide an understanding of their common experiences of their roles (Creswell 2013). Both focus group and individual interviews were carried out at a time at the venue convenient for both the researcher and the participants (Brink, van der Walt and van Rensburg 2013). Two schools were identified where two FGIs were conducted with the assistance of a trained research assistant. The main question asked was: ‘what is your role in the implementation of school-based HIV and AIDS prevention programmes?’ The techniques of probing, paraphrasing and clarification were used by the researcher throughout the interviews to elicit rich data. Each focus group was repeated thrice in a quiet room until data saturation was reached. The focus group discussions lasted for 50–55 min. In addition, in-depth individual interviews were conducted with seven PHC nurses, two NGO members and a district health manager. The in-depth individual discussions lasted for 30–50 min. Both focus group discussions and individual interviews were audiotaped with participants’ permission. An interview guide was used to guide the process. At the end of the interviews, all participants were acknowledged.

### Data analysis

Verbatim transcription of focus group discussions and individual interviews was conducted by the researcher immediately after the completion of interviews. Field notes were prepared as a critical step for data analysis whilst collecting the data. Tesch’s method of data analysis (1990) was used for the coding process (Creswell 2009). An independent coder was used to further code the data and came out with the codes, themes, categories and subcategories. The researcher met with the co-coder to compare, contrast, discuss as well as making adjustments of the data codes.

### Ethical consideration

Ethical clearance was obtained from the ethics committee of the University of Pretoria (IRB 0000 2235 IRG000176). Mpumalanga Department of Health and Mpumalanga Department of Education granted permission to conduct the study. All participants signed a consent form to indicate their willingness to take part in the study. The following principles guided the ethical considerations adhered to in the study: the right to freedom from harm and discomfort, respect for human dignity and the principle of justice.

### Trustworthiness

Ethical clearance was obtained from the ethics committee of the University of Pretoria (IRB 0000 2235 IRG000176). Mpumalanga Department of Health and Mpumalanga Department of Education granted permission to conduct the study. All participants signed a consent form to indicate their willingness to take part in the study. The following principles guided the ethical considerations adhered to in the study: the right to freedom from harm and discomfort, respect for human dignity and the principle of justice.

## Results

Three themes emerged as the roles of local role players. These roles included: provision of HIV and AIDS services, HIV and AIDS education and collaboration between role players and services. Table 1 presents themes and subthemes based on the roles of local role players in the implementation of school-based HIV and AIDS prevention programmes.

**TABLE 1:** Themes and subthemes.

Themes	Subthemes
1. Provision of HIV and AIDS health services	Youth-friendly clinic Clinic visits Screening and health education
2. HIV and AIDS education	Life orientation and health education Life skills education Information sessions
3. Collaboration between role players and services	Role players Role fulfilment

AIDS, acquired immunodeficiency syndrome; HIV, human immunodeficiency virus.

## Human immunodeficiency virus and acquired immunodeficiency syndrome health services (nurses)

Human immunodeficiency virus and AIDS health services have emerged as the foremost role for role players, with three subthemes, namely, youth-friendly clinic (YFC), clinic visits, and screening and health education. The nurse participants took provisions of HIV and AIDS health services to learners as their top priority role. It was further revealed that HIV and AIDS health services were provided in the form of visits to YFCs. Screening and health education formed part of the services and activities rendered by nurses in YFCs.

### Youth-friendly clinic

Nurses revealed that they provided youth-friendly SRHs as part of their role in implementing the school-based HIV and AIDS prevention programmes. The following statements from clinic nurses verify this finding:

‘We run a youth-friendly clinic in this clinic every day. In this clinic, we only see youth and most of them are from the nearby school.’ (Clinic participant No. 3)

‘We are having a youth-friendly clinic, which is run especially during the weekends, so we teach the youth about STI, HIV, TB and family planning.’ (Clinic participant No. 1)

‘We are having a youth-friendly clinic, which is run especially during the weekends.’ (Clinic participant No. 2)

The above statements further explain the frequency of YFCs, which were usually carried out over the weekends.

### Clinic visits

Findings from FGIs and individual interviews identified clinic visits as the platform for meeting the health needs of learners. The clinic visits by learners were either voluntary (clinic visits were learners’ preferred approach) or referrals. The visits were used for promotive, preventive and curative health services. Some of the responses are as follows:

‘We are also supposed to advise the learners to visit different clinics and hospitals where they get more information about certain diseases including HIV and AIDS.’ (Participant No. 1, FGI school A)

‘The students [*learners*] are happy and always willing to come on weekends to the clinic, because they know they will receive personalised care.’ (Clinic participant No. 4)

‘You know what, on weekends, there are less people and chances that they [*are*] seen by others or relatives, who may go out and gossip about them, is very small. They are very free.’ (CHC participant No. 1)

### Screening and health education

Participants identified screening and health education as HIV and AIDS health services to be rendered to both educators and learners. They expressed their role in relation to screening and health education as follows:

‘As nurses in this clinic, we usually screen the learners for HIV and AIDS when they came [*sic*] for any service in the clinic. We do counselling and testing for HIV … that is what we do for screening. We sometimes find learners who were [*sic*] coming for something else at the health centre, positive for HIV after we did the screening. And now with the new guidelines, we can start treatment, refer and offer support.’ (Clinic participant No. 1)

‘For all the people who came [*sic*] for family planning or any reproductive health service, there are screening tests that are done. In our case, we screen the learners and the educators … but the learners are the one [*sic*] at high risk for HIV and AIDS, so it is important that we screen them.’ (CHC participant No. 4)

Learners visiting the clinics for any health services were screened and provided health education on HIV and AIDS Counselling and Testing (HCT).

## Human immunodeficiency virus and acquired immunodeficiency syndrome education

Human immunodeficiency virus and AIDS education emerged with the following five subthemes: LO and health education; life skills education; information sessions; counselling; and school health programmes and campaigns. This education was provided by nurses, NGOs and educators on HIV and AIDS.

### Life orientation and health education

This is a formal school programme introduced in South Africa to empower learners with all skills, including sex education. Learners are guided to develop their full potential and are provided with opportunities to make informed choices regarding personal and environmental health, study opportunities and their future careers (DoE 2011).

Participants had this to say on the introduction of LO curriculum as a formal school subject:

‘Yes, I am aware of LO as an official school subject. I think it is a good subject as it helps in empowering the learners with information that will help them to make decisions related to HIV and AIDS prevention. Yes, I am aware … and I believe it is a good subject as it gives the learners information related to HIV and AIDS.’ (Clinic participant No. 1)

‘Ja [*Yes*]. ‘Am aware of it, my children share a lot of information with me, of which they tell me they learned at school during LO periods.’ (Participant No. 7, FGI school B)

All participants were aware of LO curriculum in schools and believed that it empowers learners to make sexual health decisions.

### Life skills education

Life skills education is a programme that focusses on the prevention of HIV and AIDS. This has proved to be more effective in changing learners’ behaviours because it includes a balanced approach of knowledge, skills and attitudes towards HIV and AIDS as opposed to prevention programmes that emphasised only on information. Participants said the following on life skills education:

‘We are fortunate because we do have life skills, not necessary as a subject, but as part of the curriculum whereby some of us as educators have gone for workshops. So the life skills programme helps us in, on [*sic*] how to start with such topics, because if ever you can just get into the class and talk about it, it might be difficult to be direct with the person. I can say, we just start as from the outside of the class by just asking them questions about what they see every day, what they hear every day around the HIV and AIDS [*issue*].’ (Participant No. 1, FGI school B)

‘In the case of life skills, there are topics like decision-making and being responsible when it comes to their behaviours. So such topics do help a lot, because they let the learners be aware of being responsible, and the consequences thereafter.’ (Participant No. 2, FGI school B)

Educators are capacitated to offer life skills as this makes it easier for them to tackle HIV and AIDS issues.

### Information sessions

Information sessions are opportunities given to learners by nurses, NGOs and educators to share health information. Participants alluded as follows:

‘We have information sessions with the learners occasionally. The educators and sometimes the clinic staff arrange such sessions.’ (Participant No. 1, FGI school A)

‘They are supposed to. In actual [*sic*] fact, what we normally do, we even invite [*the*] Department of Health. We had a session where they come in and, you know, speak to these learners, you know, talking to them, showing them the dangers of unprotected sex and all those things. So we still sometimes have a session, although we don’t involve the whole school, we might invite all the Grade 8 learners and then get somebody from the health department to come and talk to them.’ (Participant No. 1, FGI school B)

Sessions held in schools are conducted in collaboration with members from the DoH.

## Collaboration (referral system) between role-players and services

Collaboration means that various role players cooperated and worked jointly together for implementing the school-based HIV and AIDS prevention programmes. Participants echoed the importance of identifying and knowing all the role players and implementing the school-based HIV and AIDS prevention programmes. This was said to enhance collaboration.

### Role-players

The participants also identified the role players to be involved in implementing the school-based HIV and AIDS prevention programmes.

In identifying the role players, the participants in the study responded as follows:

‘Health promoters and who else? The members of the multidisciplinary health team, that is, the doctors in case there is a sick learner, nurses PHC and HIV courses trained, educators, parents, preachers, psychologist and social [*workers*].’ (CHC participant No. 1)

‘Except [*for*] the NGOs, I think there is this about they are also the NGOs, there is this psychologists. There are some of them, and the social workers.’ (Participant No. 10, FGI school B)

‘We have members from the Department of Health, a health promoter, the youth desk from the local police station as well as the youth centre.’ (NGO participant No. 2)

The identified role players consisted of health promoters, medical doctors, PHC nurses, HIV-trained nurses, educators, parents, church leaders, psychologists, social workers, police officers and NGO members.

### Role fulfilment

Participants felt they had to fulfil certain roles in order to implement the school-based HIV and AIDS prevention programmes. The following statements explain as to how participants perceived role fulfilment:

‘Our role, we need to focus more on giving health education.’ (CHC participant No. 4)

‘Yes, we have our own programme of which we gather information from the health centre to identify new trends in schools and then draw our programmes in relation to the information we find in the clinic. In the afternoon we go back to the facility to do our research. And on Fridays usually we meet to consolidate all the work done in our allocated schools.’

‘Yes, I feel so happy with myself as all health issues are tackled based on our research findings in the facility.’ (Clinic participant No. 2)

‘I am supposed to give health education, education peer, peer education to educate these kids about HIV and AIDS and the prevention, spread, all those issues of HIV so that they must have that information.’ (CHC participant No. 5)

‘The main role of the district manager is to monitor the efficiency and effectiveness of the programme.’ (Clinic participant No. 1)

The study participants felt that educators play the most fulfilling role in the school-based HIV and AIDS prevention programmes, as they are the primary educators of LO curriculum. The other role players came in as secondary role players. The participating nurses emphasised that they would be grateful and fulfilled if they were allowed to adequately fulfil their role in providing health education to learners. They further indicated that they also have a role to fulfil with regard to conducting research on topics or in domains relevant to the school-based HIV and AIDS prevention programmes. The participants acquiesced that they did not contribute or do enough in terms of conducting the research.

## Discussion

### Theme 1: Provision of human immunodeficiency virus and acquired immunodeficiency syndrome health services

The participants in the study emphasised that they have important roles to play in preventing HIV and AIDS in schools. They declared that in spite of working in different health and education sectors, they all shared a common goal when it came to HIV and AIDS prevention, namely, to successfully implement the HIV and AIDS prevention programmes in school settings. Various roles such as providing HIV and AIDS health services, giving HIV and AIDS education, ensuring collaboration between role players/services and monitoring and evaluation of the programmes, highlighting educators’ and nurses’ views about these programmes. The functioning of role players as identified by participants in the current study are in line with the DoH and DBE (2012) package of health services, which is provided as a bare minimum in all schools. Visiting YFCs was found to be the way to provide HIV and AIDS health services. A YFC is necessary and should function on certain principles and values such as confidentiality, respectful treatment, integrated service, culturally appropriate care, free or low-cost services and easy accession (WHO 2012). These values are necessary to be observed whilst providing healthcare service and support. Providing healthcare services that are youth-friendly is a key strategy for improving the health of the youth (Geary et al. 2014; SANAC 2017).

Shabani, Moleki and Thupayagale-Tshweneagae (2018) in their study have advocated for sexual and reproductive healthcare services (SRHCS), which is aimed at achieving and promoting both sexual and reproductive health in order to have a state of physical, mental and emotional well-being of the youth. Sexual and reproductive healthcare services are also aimed at making the services relevant for adolescents, which is the same as youth-friendly services. Health services that are not youth-friendly result in the underutilisation of such services (Ramathuba, Khoza & Netshikweta 2012). Visiting clinics is advocated because there nurses have the freedom to educate and advise learners on all areas of sexuality, STIs, HIV and AIDS, family planning as well as other areas included in the youth reproductive health services. Visiting clinics also provides an excellent opportunity for the full screening of youth and adolescents because of the prompt availability of all screening resources.

It has been highlighted in this study that nurses and NGOs visiting schools to guide students on SRHs are expected to stay within the prescripts of the school’s policy guidelines. Studies have confirmed that role players have certain limitations in functioning because of the current guidelines. The guidelines further prescribe that students are to be guided on abstinence only; condoms or any other methods to prevent the spread of HIV and AIDS as well as other STIs are not to be mentioned in schools. The real-life situation of today’s youth and the findings of the current study directly contradict schools’ policy guidelines for nurses regarding sexual and reproductive health information given to students (Hall et al. 2017).

### Theme 2: Human immunodeficiency virus and acquired immunodeficiency syndrome education

In the current study, the provision of HIV and AIDS education has been identified as one of the key aspects. It was found that through education it is possible to transmit information on HIV and AIDS regarding life skills, counselling sessions and school programmes/campaigns. Without the necessary skills, knowledge and correct attitude, the role players will not be able to render accurate and effective information. The participants commented that the role of HIV and AIDS educators requires them to have all possible knowledge needed by students an/or participants to meet the goals of programmes. This is confirmed by Borawski et al. (2015) and Hoseinpour et al. (2015), who suggested that the responsibility of imparting information must be shared between each role player and the organisation. The educators were found to be the main and the most active role players in educating and imparting knowledge to learners. Although it was a formal role to teach the subject in schools, they confirmed that the involvement of other role players with adequate knowledge on HIV and AIDS was of vital importance. The participants noted that nurses were singled out as important role players, particularly where health-related matters were concerned, because nurses had the first-hand information on health matters. These findings raised concerns regarding the medical accuracy of the information given to participants in relation to sexual and reproductive health as well as HIV and AIDS. Ma, Fisher and Kuller (2014) have raised the same concerns regarding details of the teachings. Life orientation curriculum was found to be an excellent programme meant to empower learners on HIV and AIDS and all other related issues. The concern raised in the current findings could negatively affect the intentions of DoE to introduce LO curriculum as an official school subject (DoE 2002a) if the information provided to students is not accurate medically. Furthermore, the study revealed that the amount of sex education, and information on HIV and AIDS contained in the LO curriculum is minimal to affect the desired outcome. Life skills, information sessions, school health programmes and counselling are to be used by role players to educate students on HIV and AIDS.

### Theme 3: Collaboration between role-players and services

The study highlighted the need for collaboration with and between different role players. The participants indicated that for them to effectively collaborate, they need to identify all the role players involved in the implementation of the school-based HIV and AIDS programmes. They added that role clarification for each role player should be the next step to forge meaningful and successful collaborations. The finding was further supported by ISHP’s stipulations (NDoH & DBE 2012). The ISHP emphasises that collaboration should not be limited to the healthcare teams but must be extended to all relevant role players. According to Peu (2015), collaboration must also exist between school health service and nurses. The researcher’s stance is that collaboration in school health remains the cornerstone for better education as far as teaching and learning is concerned. Furthermore, Advocates for Youth (2013) advocated for and supported thoroughly ruminated policies for school-based sexual health education and access to services. These policies must be developed collaboratively with parents, educators, administrators, students and other community members. According to Advocates for Youth (2013), this type of collaboration can ensure that the policies reflect the needs of students and the values of the community. Ultimately, the collaboration between identified stakeholders could yield a positive uptake of the programmes.

Collaboration between organisations and role players came up very strongly. The role players include, amongst others, health promoters, doctors, PHC nurses, HIV/AIDS-trained nurses, educators, parents, preachers, psychologists, social workers, police officers and NGOs. Frohlich et al. (2014) and Peu et al. (2015) affirmed that successful collaboration amongst educators, parents, DSD and DoH could yield positive results in combating the causes and effects brought about by reproductive issues. The participants shared a common belief that learners are community members taking part in the community’s activities. Thus, the inclusion and involvement of parents, church leaders and community’s social group members in the school-based HIV and AIDS prevention programmes are inevitable for it to be successful. Involvement of religious and community leaders during the process could prove to be the key to the success of these programmes in schools. Surur and Kaba (2017) supported these findings and emphasised on the proper training of these leaders and developing culture-specific and religion-specific training manuals to empower them.

## Strength and limitations

Human immunodeficiency virus and AIDS are still sensitive topics for most people because of the stigma associated with these. This was one of the limitations of the study because the participants were not comfortable to openly and freely discuss issues involving HIV and AIDS. The cultural background of participants was another serious limitation because it made them cautious about discussing some issues around these topics. In one of the schools, construction was in progress, which was noisy; it sometimes became difficult to hear participants’ words because of the noise being created outside. This resulted in some of the recordings being inaudible, making it quite difficult for the researcher to transcribe participants’ exact words during data collection. All this made data analysis quite a time-consuming exercise.

The findings of this study may not be transferrable nationally because the study was conducted in two high schools and two medical facilities only. Time was also a limitation during data collection as the participants were not available at the same time. Patients waiting in the health facilities caused some worry as the researcher could only conduct one or two interviews in a day.

## Implications of the findings

The findings of this study imply that programmes to prevent HIV infections amongst youth and adolescents could become more effective if they include a combination–prevention approach. These programmes have to be youth-friendly to promote comprehensive services that include sexuality education, knowledge of HIV, access to SRHs, and discussions on harmful sexual norms and practices. Another inference is that the programmes are to be implemented in school-based health centres/clinics (SBHCs). This is also supported by the Advocates for Youth (2013). The school-based health centres/clinics usually function from school premises or it is a mobile unit operated by a hospital or the health department. School-based health centres/clinics generally provide both primary care and mental health services to students. Sexual and reproductive health services on campuses, including but not limited to Sexual Transmitted Disease/Infection (STD/I) and HIV testing, pregnancy testing, pap and pelvic examinations, and contraceptives, could be added in these clinics to meet the youth-friendly service requirements

Nurses have to take a lead in the implementation of these programmes. Nurses have to build relationships and trust with students, parents, educators and other stakeholders. More importantly, nurses must be able to network with other role players by being involved in school activities and being up-to-date with the latest information. In addition, nurses must use social media platforms, such as Facebook, Skype and Twitter, to reach and break barriers between the involved role players.

Parents’ roles and responsibilities towards programmes are vital aspects that need attention because parents are regarded as the most important role players for the success of any programme. This includes community involvement with all other important stakeholders of the community.

Partnerships and collaborations with NGOs and other available partners involved in the prevention of HIV and AIDS must be forged to increase sponsors and sharing of resources to be used to implement programmes.

On-site comprehensive health services in high schools are seen as the way to go. This could enhance the planning and implementation of school-based HIV and AIDS prevention programmes and could allow for maximum collaboration with other role players and stakeholders involved in the programme implementation.

## Recommendations for nursing practice and nursing education

The study recommends that nurses should lead the school-based HIV and AIDS education project in high schools with the assistance of educators and NGOs. It is also recommended that nurses must be provided opportunities for community involvement and awareness campaigns to be updated on sex education to be rendered in both schools and public health services.

## Conclusion

The identified roles as stipulated by the participants of this study are widely supported by various researchers. The provisions of health services, HIV and AIDS education, and collaboration are discussed and found to be the main concerns of various stakeholders involved in the implementation of the school-based HIV and AIDS prevention programmes. The discussions revealed minimal evaluation of various programmes implemented in general. This revelation is a major concern as the impact of the programmes was not measured accurately. These themes provide the build-up for developing guidelines for local role players to implement the school-based HIV and AIDS prevention programmes.
